# Use of pedicled thigh flaps for complex abdominal wall reconstruction: A case series

**DOI:** 10.1016/j.jpra.2026.02.023

**Published:** 2026-03-06

**Authors:** Reece Hefner, Anirudh Kulkarni, Hakan Guven, John B. Park, Margaret Dalena, Alekha Kolli, Richard L. Agag, Jeremy C. Sinkin

**Affiliations:** a Division of Plastic and Reconstructive Surgery, Department of Surgery, Rutgers Robert Wood Johnson Medical School, New Brunswick, NJ 08901, USA; b Division of Plastic and Reconstructive Surgery, Department of Surgery, Beth Israel Deaconess Medical Center, Harvard Medical School, Boston, MA 02445, USA

**Keywords:** Abdominal wall reconstruction, Hernia repair, Anterolateral thigh flap, Pedicled thigh flap, Mesh reinforcement, Complex reconstruction

## Abstract

Complex abdominal wall defects that result from oncologic resection, infection, or trauma often require advanced reconstructive techniques beyond primary fascial closure or mesh reinforcement alone. In cases where primary closure is not feasible due to extensive tissue loss, pedicled thigh-based flaps offer a robust, vascularized solution to restore abdominal wall integrity. In this case series, we present three consecutive patients who underwent abdominal wall reconstruction using variations of the pedicled thigh flap based on the lateral femoral circumflex vascular pedicle, performed in conjunction with mesh reinforcement. Each patient presented with a large, complex defect following cancer resection or hernia repair complicated by prior infection or radiation. The reconstructions utilized different variations of the anterolateral thigh (ALT) flap, including fasciocutaneous, fascial, and myocutaneous components, tailored to defect size and tissue requirements. In all cases, biologic or bioresorbable mesh was incorporated to support myofascial continuity. All patients had uncomplicated recoveries, with one experiencing minor superficial wound dehiscence managed conservatively. No flap failures, donor site morbidity, or hernia recurrences were observed during follow-up periods. Our findings support the use of pedicled thigh flaps as a versatile and reliable option for complex abdominal wall reconstruction, especially when combined with mesh to optimize structural support and minimize donor site complications.

## Introduction

Plastic surgeons perform abdominal wall reconstruction due to various surgical indications including but not limited to oncologic resections, infections, hernias and trauma.^1^ Defects exist when one or more components (skin, fascia, or muscle) of the abdominal wall are missing.^2^

When possible, primary fascial closure should be performed to repair an abdominal wall that can resist stress and strain to lower rates of hernia recurrences.^3^ If the defect does not allow for primary closure, component separation is an option to achieve tension-free fascial closure.^4^^,^^5^ Mesh reinforcement via underlay or overlay is also a common technique to re-establish abdominal wall myofascial continuity; however, these techniques may be inadequate for larger, complex defects.^6^ When primary fascial approximation is not possible, pedicled or free flaps may be used for abdominal wall reconstruction.^7^^,^^8^ Specifically, thigh-based flaps based on the descending branch of the lateral circumflex femoral artery have been proven effective for reconstructing large defects while minimizing donor site morbidity.^2^

These thigh-based flaps are particularly useful for their versatility, as fascial, fasciocutaneous, or myocutaneous perforator flaps, with the option for reinnervation using the lateral femoral cutaneous nerve.^7^ This adaptability makes thigh-based flaps an ideal choice for restoring form and function to a wide range of regional and distal defects. Additionally, these flaps have successfully been used in conjunction with a biological mesh, such as in cases of enterocutaneous fistula closure (EFC), providing a robust and well-vascularized tissue layer to support healing in contaminated fields.^8^

In this case series, we present our experience using thigh-based pedicled flaps in conjunction with mesh for complex abdominal wall reconstruction.

## Methods

We identified all cases in which pedicled thigh flaps were performed at our institution for abdominal wall reconstruction by senior authors (RLA, JCS) from 09/2017 to 12/2024. Three consecutive cases were identified. We retrospectively extracted demographic, clinical, and outcomes data to present descriptions of the cases below (Tables 1 & 2). Mesh selection (biologic v. synthetic) was individualized based on patient history, cost, and infection risk.

**Table 1:** Patient demographics and defect/flap/mesh characteristics.

Case	Age	Sex	Race	Primary surgery indication	Defect location	Defect size	Flap type	Neurotized	Mesh type	Mesh layer
1	64	M	Other	Ventral hernia and fistula	Suprapubic	14 cm × 8 cm	Fasciocutaneous	No	Strattice	Underlay
2	58	F	White	Recurrent ventral hernia	Infraumbilical	10 cm × 10 cm	Fascia lata	No	Phasix	Underlay
3	41	M	Other	Metastatic rectal adenocarcinoma	Suprapubic to umbilical	10 cm × 12 cm	Chimeric muscle and fasciocutaneous	Yes	Strattice	Bridging

**Table 2:** Postoperative variables.

Case	Hospital LOS	Reoperation	Readmission	Donor site complications	Recipient site complications	Hernia recurrence	Length of follow-up (Days)
1	19	No	No	No	Superficial dehiscence	No	104
2	5	No	No	No	No	No	78
3	8	No	No	No	No	-	67

### Case 1

65-year-old male with advanced rectosigmoid cancer initially treated with a diverting loop colostomy, radiation, and chemotherapy presented with colocutaneous fistula through his anterior abdominal wall. After resection of the fistula, the patient had an open lower pelvic abdominal wound measuring 8 × 14 cm. The heavily contaminated defect was temporized with a bridging porcine biologic mesh (Strattice). Three days after the primary surgery, the plastic surgery team reconstructed the defect with a pedicled fasciocutaneous anterolateral thigh (ALT) flap. The patient had an uncomplicated recovery with a hospital stay of 19 days. Postoperatively, he developed a minor superficial flap dehiscence measuring 2 × 2 cm managed with local wound care. No other complications were noted. Patient had a follow-up period of 104 days.

### Case 2

58-year-old female presented for repair of recurrent ventral hernia following multiple prior operative repairs complicated by mesh infection. Following reduction of the abdominal hernia by general surgery and removal of the infected mesh, the patient had a 10 × 10 cm midline fascial defect. Due to the patient's history of previous mesh infection and poor quality of abdominal fascia, this defect was closed primarily and reinforced with an underlay P4HB (Phasix ST) mesh followed by further reinforcement with a pedicled ALT flap. The patient had an uncomplicated hospital course and was discharged on postoperative day 5. No complications or hernia recurrence were reported at 78-day follow-up.

### Case 3

**Figure 1:**
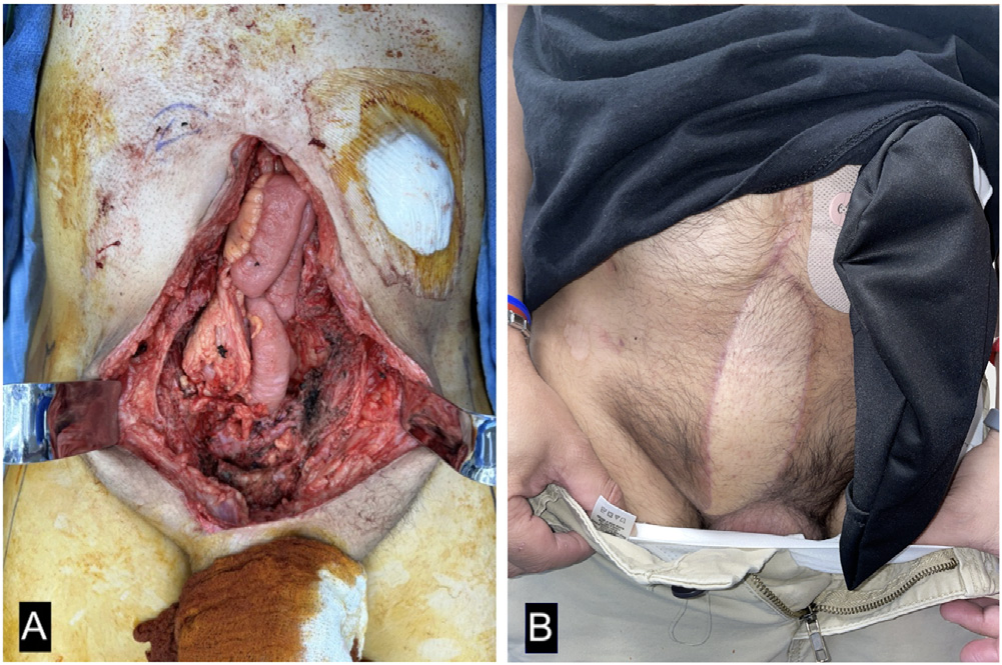
Images of case 3. (A) Intraoperative evaluation of defect after resection. (B) Evaluation at 67-day follow-up.

41-year-old male with anorectal cancer with history of abdominoperineal resection and chemoradiation presented with cutaneous metastases to the lower abdominal and pelvic wall. Patient underwent resection, involving the anterior portion of the bladder and resulted in a 10 × 12 cm full-thickness defect of the abdominal wall with loss of the right rectus abdominis muscle, posterior rectus sheath, with extension into the abdominal cavity (Figure 1A). Due to the extensive resection, a neurotized ALT pedicled myocutaneous flap with a bridging Strattice was used to restore abdominal domain and reconstruct myofascial unit (Figure 2A and B online supplementary images). The patient had an uncomplicated recovery and was discharged on postoperative day 8. No complications were noted at 67-day follow-up (Figure 1B).

## Discussion

The patients in our case series experienced significant alteration to native abdominal anatomy secondary to extensive resections. A pedicled thigh flap with variable tissue components based on branches of the lateral femoral circumflex vasculature was used in each case to facilitate and reinforce closure of large defects. The overall average hospital length of stay among our three patients was 10.7 days.

In each of our cases, the abdominal wall flap closure was augmented by a biologic or bioresorbable mesh with effective and safe outcomes. In previous studies, EFC closure rates after resection and reconstruction with the use of both ALT and mesh were reported to be 100%, illustrating the efficacy of this approach.^8^ This case series shows similar findings to the favorable long-term complications seen in literature in treatment of EFC, with rates of dehiscence or hernia formation occurring only in a small group of patients.^8^ In our study the only postoperative complication that arose between the three patients was superficial wound dehiscence, in Case 1, which resolved appropriately with routine local wound care. The patient in Case 1 had previously undergone local radiation therapy and thus was already at an elevated risk for wound healing complications. Overall complications were minimal with the other patients having no reported complications postoperatively.

The versatility of thigh-based donor site to supply well-vascularized, composite tissue and the ability to use the flap to augment more traditional reconstruction such as mesh based abdominal wall reconstruction demonstrates how thigh-based flaps can be an invaluable asset in both primary reconstruction and revision procedures. Additionally, the benefit of limiting potential donor site morbidity when a smaller flap is taken and used in conjunction with mesh has been previously described.^2^ The results of this study are concurrent with this understanding as none of the cases experienced donor site complications or morbidity.

In situations where component separation or mesh use is not possible, the pedicled thigh flap from the lateral femoral circumflex vessels can be considered in the appropriate patient. This is especially true when primary surgery involves extensive skin removal or the potential of a contaminated field. Previous studies in conjunction with our findings support the understanding that the pedicled ALT flap offers a robust repair with a decreased risk of future eventration and can mitigate concerns of infection, wound healing complications, and recurrent herniation often associated with the risk of a bridging mesh alone.^2^

While further research is required given the limited number of published patient cases, the initial data along with the success of our own series demonstrates promise in using thigh-based flaps as a viable solution to complex abdominal wall reconstruction. It is important to note that the significance of our conclusions is limited by the small sample of patient cases examined. Additional longitudinal and more robust studies would be useful in assessing these outcomes further.

## Conclusion

In this small case series, we demonstrated pedicled thigh-based flaps are a viable and safe choice for reconstruction of complex abdominal wall defects. It offers many advantages and can be concurrently used with other methods commonly employed for closure, such as mesh reinforcement.

## Funding

None.

## Declaration of competing interest

None declared.

## Appendix Supplementary materials

Figure 2. Online supplementary images. Images of Case 3. (A) Intraoperative mesh insert. (B) Intraoperative partial flap insert. Image, image 1 dummy alt text
