# Supplementary figures and images for: Use of pedicled thigh flaps for complex abdominal wall reconstruction: A case series
Source: JPRAS Open. 2026 Mar 6;49:306–11. doi: 10.1016/j.jpra.2026.02.023 (PMC13019955; doi:10.1016/j.jpra.2026.02.023)

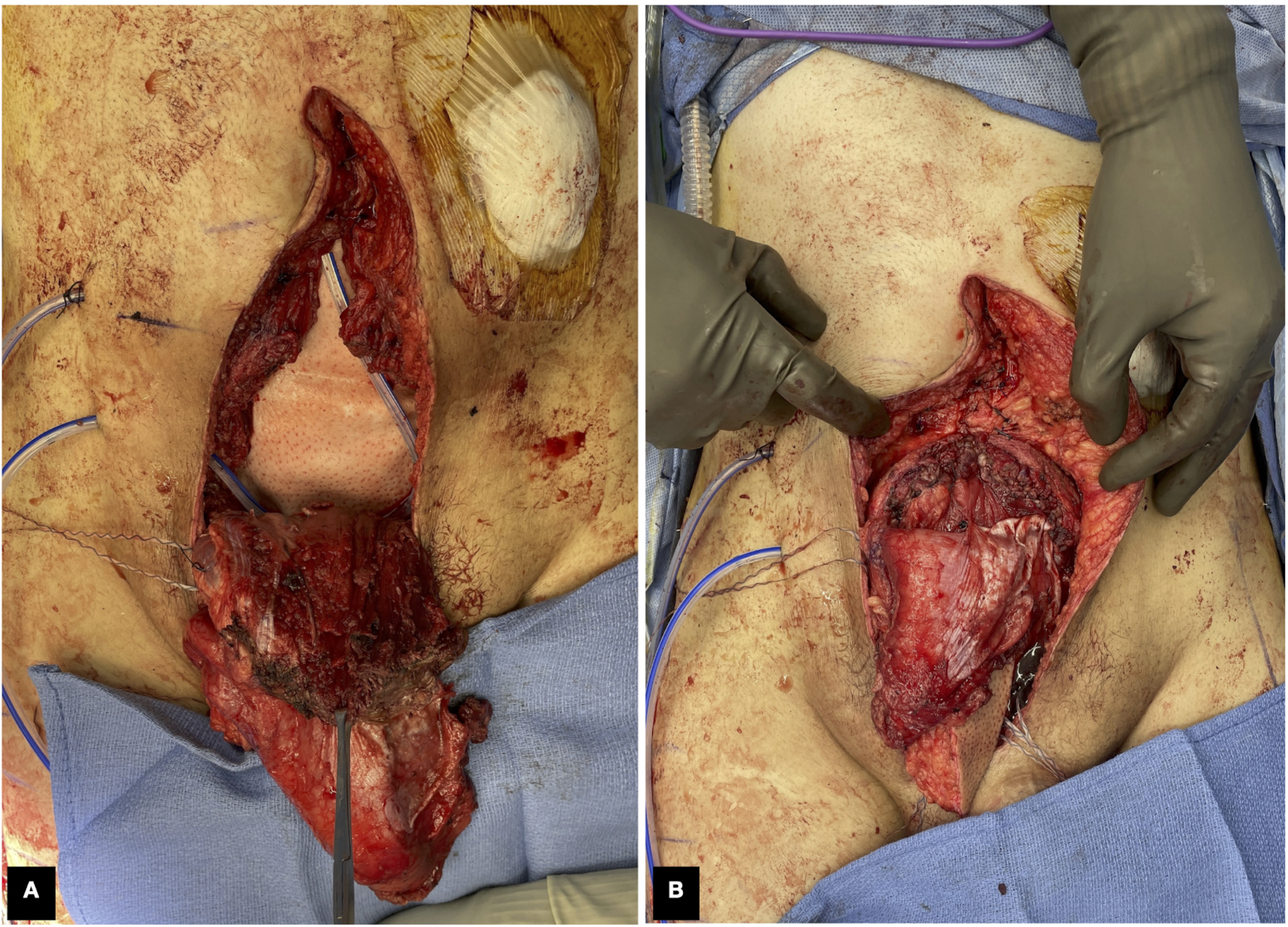

Supplement: Supplementary file 1 — Figure 2. Online supplementary images. Images of Case 3. (A) Intraoperative mesh insert. (B) Intraoperative partial flap insert. [file mmc1.jpg]
